# Editorial: Human coronavirus research: 20 years since the SARS-CoV outbreak

**DOI:** 10.3389/fmicb.2022.1035267

**Published:** 2022-09-23

**Authors:** Burtram C. Fielding

**Affiliations:** Department of Medical Biosciences, Faculty of Natural Sciences, University of the Western Cape, Bellville, South Africa

**Keywords:** severe acute respiratory syndrome (SARS-CoV), Middle East respiratory syndrome (MERS-CoV), SARS-CoV-2 (COVID-19), human coronavirus (HCoV), respiratory viral infections

This Editorial introduces 10 articles published in a Special Issue highlighting human coronavirus (hCoV) research on the twentieth anniversary of the outbreak of severe acute respiratory syndrome (SARS) in late 2002. Only with the SARS outbreak was the pandemic potential of hCoVs acknowledged. HCoV-OC43 (Tyrrell and Bynoe, 1966), HCoV-229E (Hamre and Procknow, 1966), HCoV-NL63 (Van Der Hoek et al., 2004), and HCoV-HKU1 (Woo et al., 2005) are endemic in the human population and are mainly associated with mild, self-limiting “common cold” illnesses annually. The burden of respiratory tract infections, caused by the four “common-cold” hCoVs, is increased in patients with chronic co-morbidities or clinical risk factors including young children, the elderly and immunocompromised (Van Der Hoek, 2007). On the other hand, the three know pathogenic hCoVs, SARS-CoV (Drosten et al., 2003; Peiris et al., 2003), Middle East Respiratory Syndrome CoV (MERS-CoV) (Zaki et al., 2012), and SARS-CoV-2 (Zhou et al., 2020a,b), cause severe respiratory syndromes and result in high morbidities and mortalities, especially in the elderly (Chen et al., 2020).

The current pandemic has shown that we have not effectively used the vast knowledge gained from decades of HCoV research in studying and fighting SARS-CoV-2; this has often resulted in delays in reporting on therapeutics, control measures, etc. For this Special Issue we invited contributions of human coronavirus research, with particular focus on, but not limited to, morbidity and mortality numbers, genomic differences, their distinct immune evasion mechanisms, viral-host interactions, the development of potential broad-spectrum antiviral or therapeutics.

In the first article, Teng et al. sets out to identify the aetiological agent of respiratory infections in a herd of nine dromedary camel calves in the United Arab Emirates. Using cell culture and molecular biology techniques, they identify that MERS-CoV and a dromedary camel bovine parainfluenza virus 3 (DcPIV3), a novel species of the genus *Respirovirus*, are co-circulating in this herd. This is the first report of a novel respirovirus in sick dromedaries.

In the second article, Blumenthal et al. report an observational study of adults admitted to a public hospital in South Africa during June to August 2020 tested for SARS-CoV-2 infection. The authors also measure Kaposi's sarcoma-associated herpesvirus (KSHV) serology, as well as Epstein-Barr virus (EBV) and KSHV viral load in peripheral blood, and relates it to COVID-19 prognosis. Even though the study design does not allow the authors to conclude that disease synergy exists between COVID-19 and KSHV, their data allude to a relationship between KSHV infection and COVID-19 outcome, as well as SARS-CoV-2 infection and KSHV reactivation. These findings warrant further study in countries with high disease burdens. In the article by Lau et al., a case of fatal primary pneumonia in a 75-year old patient, with good past health, is linked to a novel HCoV-OC43. With fatal primary pneumonia due to HCoV-OC43 infection rarely reported (Van Der Hoek, 2007), the authors report high HCoV-OC43 loads in the lower respiratory tract throughout the illness of the patient. They hypothesize that a four-amino-acid insertion in the immediate downstream region of the S1/S2 cleavage site is linked to the hypervirulence of this novel genotype.

In a small cohort study, using sera collected during, as well as post-, HCoV-HKU1 infection, Sechan et al. report that a substantial portion of people infected with HCoV-HKU1 display no rise in viral-specific antibodies. This finding differs from previous reports for HCoV-OC43, HCoV-NL6 and HCoV-229E, that show more typical antibody dynamics (Sastre et al., 2011). The authors hypothesize that this difference is a result of the lower disease severity of HCoV-HKU1 infections. In another antibody study, this time for SARS-CoV-2, Ren et al. look at the kinetics of secretory IgA in the mucosa of the respiratory system and non-secretory IgA in the blood of 28 COVID-19 patients and 55 COVID-19-vaccine recipients. The authors suggest that anti-NP IgA detection in sputum and throat swabs could have diagnostic value for COVID-19 prevention and control. Interestingly, in their article, Irungbam et al. report evaluating the diagnostic efficacy of rapid IgM and IgG chemiluminescence kits targeting the receptor-binding domain (RBD) of the SARS-CoV-2 spike protein in a real-world hospital setting in India. They conclude that SARS-CoV-2 diagnostic tools using the detection of anti-RBD IgM are less effective than diagnostic tools using anti-RBD IgG in a real-world hospital setting.

Xie et al. report the generation of a mouse-adapted HCoV-OC43 strain, VR-1558, that is effective and reproducible for anti-HCoV-OC43 drug studies. Following an antiviral drug screen, they report that arbidol hydrochloride and Qingwenjiere Mixture decrease symptoms and improve the survival rate of infected mice. The authors report that the two compounds result in a measurable decrease in the expression of N, the inflammatory response and pathological changes. In another article, reporting the database screening of more than 3,400 compounds, Matos et al. identify Hypericin as one of the top candidates with high binding affinity to viral Mpro and RdRp. With Mpro and RdRp known to be critical for coronavirus replication (Stobart et al., 2012), the authors then use cell culture studies to show that Hypericin has anti-SARS-CoV-2 properties. This study is the first step in establishing the suitability of Hypericin as an anti-SARS-CoV-2, and possibly a broad-spectrum anti-coronaviral, drug. Zhang et al. report in their article that the SARS-CoV-2 nucleocapsid protein (N) melts double-stranded DNA (dsDNA) in the 5′-3′ direction. Moreover, the authors hypothesize that the protein's ssDNA binding and dsDNA unwinding activity show that the N binds to the host's genomic ssDNA during replication and affects host cell replication. These findings could also inform the future development of broad-spectrum anti-coronaviral agents.

In the final article, showing the multidisciplinary approach needed to understand and fight coronavirus pandemics, Mehta et al. review recent studies to understand the interplay of nutrition and psychoneuroendocrineimmune (PNEI) modulation, and its impact on the prognosis and convalescence of COVID-19. The authors report that large population sizes, high prevalence of undernourishment, and high incidence of mental health issues increase the risk for COVID-19. They conclude that the monitoring of these factors will assist in designing complimentary pandemic interventions through medical nutrition therapy and psychopsychiatric management.

The COVID-19 pandemic has showed us that the human coronaviruses pose a real threat. It has taken us 56 years since the discovery of the first human coronaviruses, and millions of deaths (Figure 1) to develop the first human coronavirus vaccine. This Special Issue provides a platform to share the work done on the known hCoVs, and should provide us with a better research foundation when the next pathogenic hCoV is identified.

**Figure 1:**
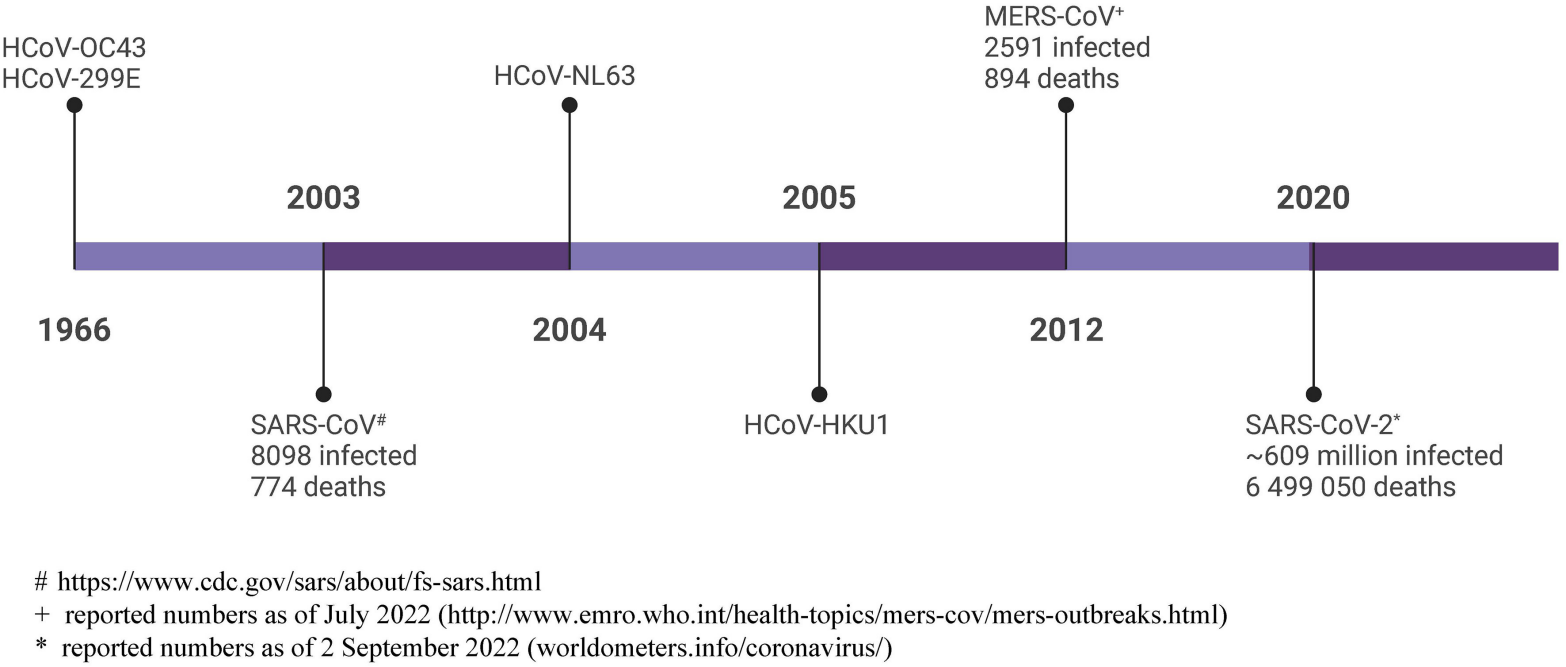
Timeline of human coronavirus discovery, with total reported infections and deaths shown. # https://www.cdc.gov/sars/about/fs-sars.html; + reported numbers as of July 2022 (http://www.emro.who.int/health-topics/mers-cov/mers-outbreaks.html); ^*^ reported numbers as of 2 September 2022 (worldometers.info/coronavirus/). Created with BioRender.com.

## Author contributions

This editorial was written solely by the one guest editor.

## Conflict of interest

The author declares that the research was conducted in the absence of any commercial or financial relationships that could be construed as a potential conflict of interest.

## Publisher's note

All claims expressed in this article are solely those of the authors and do not necessarily represent those of their affiliated organizations, or those of the publisher, the editors and the reviewers. Any product that may be evaluated in this article, or claim that may be made by its manufacturer, is not guaranteed or endorsed by the publisher.
